# Factors of psychiatric emergencies affecting boarding time in the emergency department

**DOI:** 10.1192/j.eurpsy.2021.965

**Published:** 2021-08-13

**Authors:** K.B. Avanoğlu, Ş. Gürel

**Affiliations:** Psychiatry Department, Hacettepe University Hospitals, Ankara, Turkey

**Keywords:** Psychiatric Emergency Services, Quality of Care, Suicide, psychosis

## Abstract

**Introduction:**

Psychiatric emergencies are acute disturbances in thought, behavior or mood which require immediate medical intervention. As a substantial number of patients with mental illness present as psychiatric emergencies, the sustainability and management of psychiatric emergency services becomes significant.

**Objectives:**

In this study we aimed to examine the factors associated with psychiatric emergency care, taking the boarding time in the emergency department as primary outcome measure.

**Methods:**

Charts of 466 psychiatric emergency cases admitted to the Hacettepe University Emergency Department (ED) between December 2018 – September 2019 were evaluated. Boarding time (BT) in the ED, presence of self-harm, psychotic symptoms and agitation were noted.

**Results:**

In the examined period, number of patients admitted increased with time significantly (r= 0.562, p <0.01). However, increase in the number of patients was not correlated with an increase in BT. Patients with psychotic symptoms had greater BT compared to non-psychotic patients (7.01 hours vs. 11.24 hours, T= -2.796 df = 182.717 p <0.01). Patients with self-harm also had greater BT (7.47 hours vs. 9.85 hours, T = -2.013 df = 433 p <0.05). Patients with self-harm in relation with previous suicidal ideation displayed significantly a longer BT when compared with patients admitted due to self-harm without any suicidal plan (U=2572,5 p<0.01).

**Conclusions:**

A significant increase in BT with psychosis and self-harm due to a suicidal plan supports the need of intermediate facilities between the ED and inpatient units, as such facilities would create a positive impact in the care of psychotic and suicidal patients.

